# Association between immunoglobulin G N-glycosylation and lupus nephritis in female patients with systemic lupus erythematosus: a case-control study

**DOI:** 10.3389/fimmu.2023.1257906

**Published:** 2023-09-21

**Authors:** Xinxia Lu, Liangao Wang, Meng Wang, Yuejin Li, Qinqin Zhao, Yanjun Shi, Yujing Zhang, Yingjie Wang, Wei Wang, Long Ji, Haifeng Hou, Dong Li

**Affiliations:** ^1^School of Public Health, Shandong First Medical University & Shandong Academy of Medical Sciences, Jinan, China; ^2^Jinshan District Center for Disease Control and Prevention, Shanghai, China; ^3^Shandong Institute of Parasitic Diseases, Shandong First Medical University & Shandong Academy of Medical Sciences, Jining, China; ^4^Department of Geriatric Cognitive Medicine, The Affiliated Taian City Central Hospital of Qingdao University, Taian, China; ^5^Department of Rheumatology and Immunology, Liaocheng People’s Hospital, Liao’cheng, China; ^6^Centre for Precision Health, School of Medical and Health Sciences, Edith Cowan University, Perth, WA, Australia; ^7^College of Sports Medicine and Rehabilitation, Shandong First Medical University & Shandong Academy of Medical Sciences, Tai’an, China; ^8^Department of Gastroenterology, The Second Affiliated Hospital of Shandong First Medical University, Tai’an, China; ^9^Clinical Research Center, The Second Affiliated Hospital of Shandong First Medical University, Tai’an, China

**Keywords:** immunoglobulin G, N-glycosylation, lupus nephritis, inflammation, biomarkers

## Abstract

**Background:**

Lupus nephritis (LN) is a crucial complication of systemic lupus erythematosus (SLE) and has important clinical implications in guiding treatment. N-glycosylation of immunoglobulin G (IgG) plays a key role in the development of SLE by affecting the balance of anti-inflammatory and proinflammatory responses. This study aimed to evaluate the performance of IgG N-glycosylation for diagnosing LN in a sample of female SLE patients.

**Methods:**

This case-control study recruited 188 women with SLE, including 94 patients with LN and 94 age-matched patients without LN. The profiles of plasma IgG N-glycans were detected by hydrophilic interaction chromatography with ultra-performance liquid chromatography (HILIC-UPLC). A multivariate logistic regression model was used to explore the associations between IgG N-glycans and LN. A diagnostic model was developed using the significant glycans as well as demographic factors. The performance of IgG N-glycans in the diagnosis of LN was evaluated by receiver operating characteristic (ROC) curve analysis, and the area under the curve (AUC) and its 95% confidence interval (CI) were calculated.

**Results:**

There were significant differences in 9 initial glycans (GP2, GP4, GP6, GP8, GP10, GP14, GP16, GP18 and GP23) between women with SLE with and without LN (*P* < 0.05). The levels of sialylated, galactosylated and fucosylated glycans were significantly lower in the LN patients than in the control group, while bisected N-acetylglucosamine (GlcNAc) glycans were increased in LN patients (*P* < 0.05). GP8, GP10, GP18, and anemia were included in our diagnostic model, which performed well in differentiating female SLE patients with LN from those without LN (AUC = 0.792, 95% CI: 0.727 to 0.858).

**Conclusion:**

Our findings indicate that decreased sialylation, galactosylation, and core fucosylation and increased bisecting GlcNAc might play a role in the development of LN by upregulating the proinflammatory response of IgG. IgG N-glycans can serve as potential biomarkers to differentiate individuals with LN among SLE patients.

## Introduction

Systemic lupus erythematosus (SLE) is a chronic inflammatory disease that occurs more frequently in women than in men (1). SLE induces severe disorders in the skin, mucous membranes, kidneys, brain, and skeletal system, among which the kidneys are primarily affected (2). Approximately 50%-70% of SLE patients are expected to develop lupus nephritis (LN) within 5 years (3, 4). Although the mechanism underlying LN pathogenesis remains unclear, it is closely related to autoantibodies and the formation and deposition of immune complexes and is impacted by the interaction of multiple factors, including the environment, genetics and estrogen (5).

In patients with impaired immune functions, both exogenous and endogenous antigens trigger the proliferation and activation of B lymphocytes (B cells), leading to the production of various autoantibodies against organ and tissue components (6). These autoantibodies bind to their corresponding antigens, forming circulating immune complexes or *in situ* immune complexes that ultimately deposit in the glomeruli (7). Immune complexes can activate complement, triggering an inflammatory response involving multiple inflammatory factors and cells, resulting in renal damage (8, 9). Currently, renal biopsy is the gold standard for LN diagnosis; however, it has limitations in terms of invasiveness, high cost, complexity in disease histopathological grading, and inability to continuously monitor disease progression (10). Because early diagnosis and treatment of LN patients lead to a better prognosis (11), reliable noninvasive biomarkers are needed to improve early diagnosis and outcome prediction of LN (12).

Glycosylation, a crucial posttranslational modification, participates in almost all vital physiological and biochemical activities, including cell communication, adhesion, differentiation, and proliferation (13). Immunoglobulin G (IgG), one of most important glycoproteins, is the predominant antibody in the blood, accounting for approximately 75% of the total peripheral blood immunoglobulins (14). The IgG molecule contains a conserved N-glycosylation site at the asparagine (Asn) 297 position on each of its two fragment crystallizable heavy (Fc) chains (15). Fc N-glycosylation stabilizes the structure of the Fc fragment and regulates the anti-inflammatory and proinflammatory functions of IgG (16) through the following pathways: antibody-dependent cell cytotoxicity (ADCC), complement-dependent cytotoxicity (CDC), antibody-dependent cellular phagocytosis (ADCP), and other types of receptor-mediated immune regulation (17, 18). Increased core fucosylated and sialylated glycans promote the binding affinity of IgG with the activated receptor FcγRIIIa, which mediates ADCC, thus initiating activation signals and upregulating inflammatory responses (19). In addition, galactosylated glycans promote downstream CDC reactions by binding to complement 1q (C1q) (20). Proinflammatory changes in the IgG N-glycome are associated with increased levels of bisecting N-acetylglucosamine (GlcNAc) (21, 22). IgG N-glycosylation has been identified as a potential biomarker of a variety of diseases, including chronic diseases, inflammatory diseases, neurodegenerative diseases, autoimmune diseases, and cancer, offering great research potential (23–25). On the basis of our findings in the association between the IgG N-glycome and SLE patients (26, 27), we hypothesize that IgG N-glycosylation has potential value in the early diagnosis of LN among SLE patients.

## Materials and methods

### Study sample

This case-control study recruited 188 SLE patients (94 patients with LN and 94 age-matched patients without LN) from the Liaocheng People’s Hospital between July 2020 and September 2021. The inclusion criteria were as follows: (1) Chinese Han ethnicity; (2) female; (3) diagnosis of SLE and (4) SLE patients with LN who met the diagnostic criteria for LN. The exclusion criteria were (1) the coexistence of other autoimmune diseases; (2) primary glomerular diseases or secondary renal diseases; (3) severe trauma, malignant tumors, and severe infections; (4) People who have taken glucocorticoids or immunosuppressants within a month and (5) incomplete patient clinical data. A total of 94 age-matched SLE patients without LN were included in the control group, who newly diagnosed SLE and have not received corresponding treatment. The study was conducted under the supervision of the Ethics Committee of Shandong First Medical University (approval number: 201712). The study was conducted with the written informed consent of all participants.

### Diagnosis of systemic lupus erythematosus

The current diagnosis of SLE was based on the “American College of Rheumatology (ACR) in 2019” guidelines, which requires a patient to present with 4 out of 11 symptoms/disorders (28), including malar rash, discoid erythema, photic hypersensitiveness, mouth ulcer, joint inflammation, pericarditis or pleurisy, urinary protein > 0.5 g/24 h or +++, seizures or psychosis, hematological diseases, anti-Sm antibody and dsDNA antibody positivity, and antinuclear antibody positivity.

### Diagnosis of lupus nephritis

The diagnosis of LN in this study was based on the “Guidelines for the Diagnosis and Treatment of Lupus Nephritis in China” (29). The diagnosis of LN mainly included three criteria: ① persistent urine protein >0.5 g/24 hours, random urine protein +++ or a urine protein-to-creatinine ratio >50 mg/mmol; ② active urinary sediment (excluding urinary tract infection) with >5 white blood cells per high-power field, >5 red blood cells per high-power field, red blood cell casts, or white blood cell casts; ③ cellular casts including red blood cell casts, granular casts, hemoglobin casts, tubular casts, or mixed casts. When SLE patients exhibit any of the above clinical and laboratory abnormalities, a diagnosis of LN was made.

### Immunoglobulin G N-glycan analysis

We detected plasma IgG N-glycan profiles using hydrophilic interaction chromatography (HILIC)-ultra-performance liquid chromatography (UPLC) (Waters Corporation, Milford, MA, United States), which was described in a previous study (30). Briefly, the method involved the following steps: (1) plasma IgG was separated with a 96-well Protein G extraction plate (CIM^®^ r-Protein G 0.2 ml Monolithic 96-well Plate, BIA Separations); (2) IgG Fc N-linked glycans were released by PNGase F enzyme (150 units, Roche); (3) the released IgG N-glycans were labeled with the fluorescent dye 2-aminobenzamide (2-AB, Sigma); and (4) fluorescently labeled N-glycans were detected and analyzed by HILIC-UPLC. The HILIC-UPLC approach initially obtained 24 glycan peaks (GP1-GP24) corresponding to a distinct glycan structure. Composition and structure of IgG initial glycans are available in the **Supplementary Table S1** (31). An effective method for normalizing glycan measurements across samples is to divide the peak areas of each glycan by the total areas of the respective chromatograms (32).Typical chromatograms for LN and non-LN samples are provided in the **Supplementary Figure S1** (33). From the 24 initial GPs, 17 derived traits were calculated and the calculation formulas can be found in the **Supplementary Table S2** (34), which represented the relative abundances of four glycosylation features: core fucosylated, sialylated, bisected GlcNAc, and galactosylated IgG N-glycans (35). Among them, the level of aGal/Gal ratio was calculated from the relative intensity of agalactosylated (G0) vs. monogalactosylated (G1) and digalactosylated (G2) fucosylated biantennary glycans according to the formula of G0/(G1 + G2×2) as we previously described.

### Assessment of covariates

A blood sample was obtained from the large antecubital vein of each participant through venipuncture in the morning after an overnight fast. The collected sample was divided into two tubes for further analysis. The sample collected in a vacuum tube containing ethylenediaminetetraacetic acid (EDTA) underwent plasma separation to detect IgG N-glycans, while the sample collected in a tube without EDTA underwent serum separation to determine inflammatory factors and blood biochemical indices.

Face-to-face interviews, a series of clinical examinations and laboratory tests were used to collect basic patient information. Demographic characteristics such as age, sex, and the participants’ history of hypertension, diabetes, and dyslipidemia were collected. Body mass index (BMI) was calculated using measurements of height and weight, with the formula weight (kg)/height^2^ (m^2^). Hypertension was defined as the use of antihypertensive medication, a self-report of a history of hypertension, and diastolic blood pressure (DBP) ≥ 90 mmHg or systolic blood pressure (SBP) ≥ 140 mmHg. Diabetes was defined as the current use of insulin or oral hypoglycemic agents, a history of diabetes, or a fasting blood glucose level ≥ 7.0 mmol/L (126 mg/dL). Dyslipidemia was defined as the current use of lipid-lowering therapy; a self-report of a history of dyslipidemia; or serum triglycerides (TGs) ≥ 1.7 mmol/L, total cholesterol (TC) ≥ 5.18 mmol/L, high-density lipoprotein cholesterol (HDL-C) < 1.04 mmol/L, or low-density lipoprotein cholesterol (LDL-C) ≥ 3.37 mmol/L, according to the guidelines for the prevention and control of dyslipidemia in adults in China (36).

### Statistical analyses

Continuous variables with normal distributions are expressed as the mean ± standard deviation (SD) and were compared using Student’s t test. Nonnormally distributed data are presented as the median and interquartile range (IQR) and were compared with the Kruskal-Wallis rank-sum test. Categorical variables are presented as n (%) and were evaluated with the chi-square test or *Fisher’s* exact test. A multivariate logistic regression model was used to evaluate the association of influencing factors with LN, by which the odds ratio (OR) and its 95% confidence interval (CI) were calculated. In logistic regression analysis, the IgG N-glycan measurements were categorized into four groups: Q1 (values ≤ P25), Q2 (P25 < values ≤ P50), Q3 (P50 < values ≤ P75), and Q4 (values > P75). Receiver operating characteristic (ROC) curve analysis was employed to evaluate the performance of the diagnostic models.

The statistical analyses were performed using SPSS 25.0 (IBM, Armonk, NY, United States) and R software (version 3.4.3, R Core Team). All statistical tests were two-sided, and the significance level was set as *P* < 0.05.

## Results

### Baseline characteristics of the study participants

This study included 94 SLE women with LN (mean age 40.10 ± 12.86 years) and 94 age-matched non-LN patients (mean age 41.06 ± 11.83 years). As shown in **Table 1**, no statistically significant difference in age was detected between the LN group and the control group. Compared with the control group, LN patients had significantly lower serum white blood cell (WBC) counts and significantly higher levels of C3. In addition, the prevalence of anemia was significantly higher in LN patients than in controls.

**Table 1:** Characteristics of the study participants.

Characteristics	LN group (n=94)	Non-LN group (n=94)	*t*/χ²/*Z*	*P*
Age (years)	40.10 ± 12.86	41.06 ± 11.83	0.534	0.594
BMI (kg/m²)	24.22 ± 3.27	23.87 ± 3.00	0.765	0.445
WBC count (×10¹²/L)	6.79 (5.00, 8.70)	5.97 (4.29, 7.66)	2.300	0.021*
BUN (mmol/L)	4.46 (3.48, 5.82)	4.73 (3.60, 6.43)	0.907	0.364
UA (µmol/L)	250 (190, 304)	266 (197, 320)	1.045	0.296
CHOL (mmol/L)	4.78 (3.68, 5.68)	4.66 (4.08, 5.59)	0.042	0.966
TG (mmol/L)	1.41 (0.98, 1.91)	1.31 (0.99, 1.96)	0.267	0.790
HDL-C (mmol/L)	1.25 (1.02, 1.59)	1.25 (1.06, 1.57)	0.222	0.824
LDL-C (mmol/L)	2.76 (2.14, 3.39)	2.62 (2.18, 3.26)	0.503	0.615
uCRE (µmol/L)	55.7 (46.50, 63.85)	55.3 (46.00, 64.85)	0.150	0.881
eGFR (ml/min)	107.58 (88.66, 123.98)	108.42 (90.23, 121.94)	0.334	0.739
C3 (g/L)	0.66 (0.48, 0.81)	0.74 (0.58, 0.89)	2.010	0.044*
C4 (g/L)	0.14 (0.08, 0.20)	0.15 (0.10, 0.21)	0.544	0.586
Hs-CRP (mg/L)	6.25 (2.57, 14.40)	7.72 (3.53, 15.02)	0.790	0.430
IgG (g/L)	15.20 (11.55, 19.10)	15.40 (13.10, 20.30)	1.205	0.228
Anemia (n, %)	42 (45.2)	23 (24.7)	8.537	0.003*
Hypertension (n, %)	7 (7.5)	3 (3.2)	1.691	0.193
Diabetes (n, %)	10 (10.8)	12 (12.9)	0.206	0.650
Dyslipidemia (n, %)	70 (75.3)	62 (66.7)	1.670	0.196
SLEDAI-2K	4.92 ± 1.87	4.63 ± 1.57	1.147	0.252
Positive of anti-dsDNA (n, %)	42 (45.2)	48 (51.6)	0.775	0.379

*Statistically significant, P < 0.05. P values were calculated by the independent-sample t test or chi-square test and Mann-Whitney U test. BMI, body mass index; BUN, blood urea nitrogen; C3, complement 3; C4, complement 4; CHOL, cholesterol; eGFR, estimated glomerular filtration rate; HDL-C, high-density lipoprotein cholesterol; hs-CRP, high-sensitivity C-reactive protein; IgG, immunoglobulin G; IgG, immunoglobulin G; LDL-C, low-density lipoprotein cholesterol; LN, lupus nephritis; positive anti-dsDNA, positive double-stranded DNA; uCRE, urine creatinine; TG, total triglycerides; UA, uric acid; WBC, white blood cell; SLEDAI-2K, SLE disease activity index 2000.

A total of 24 initial glycan peaks (GP1 - GP24) were obtained from all the chromatograms of UPLC. The distribution of the initial glycans between the LN group and the control group was listed in **Figure S2**.

### Galactosylation of IgG

**Figure 1:**
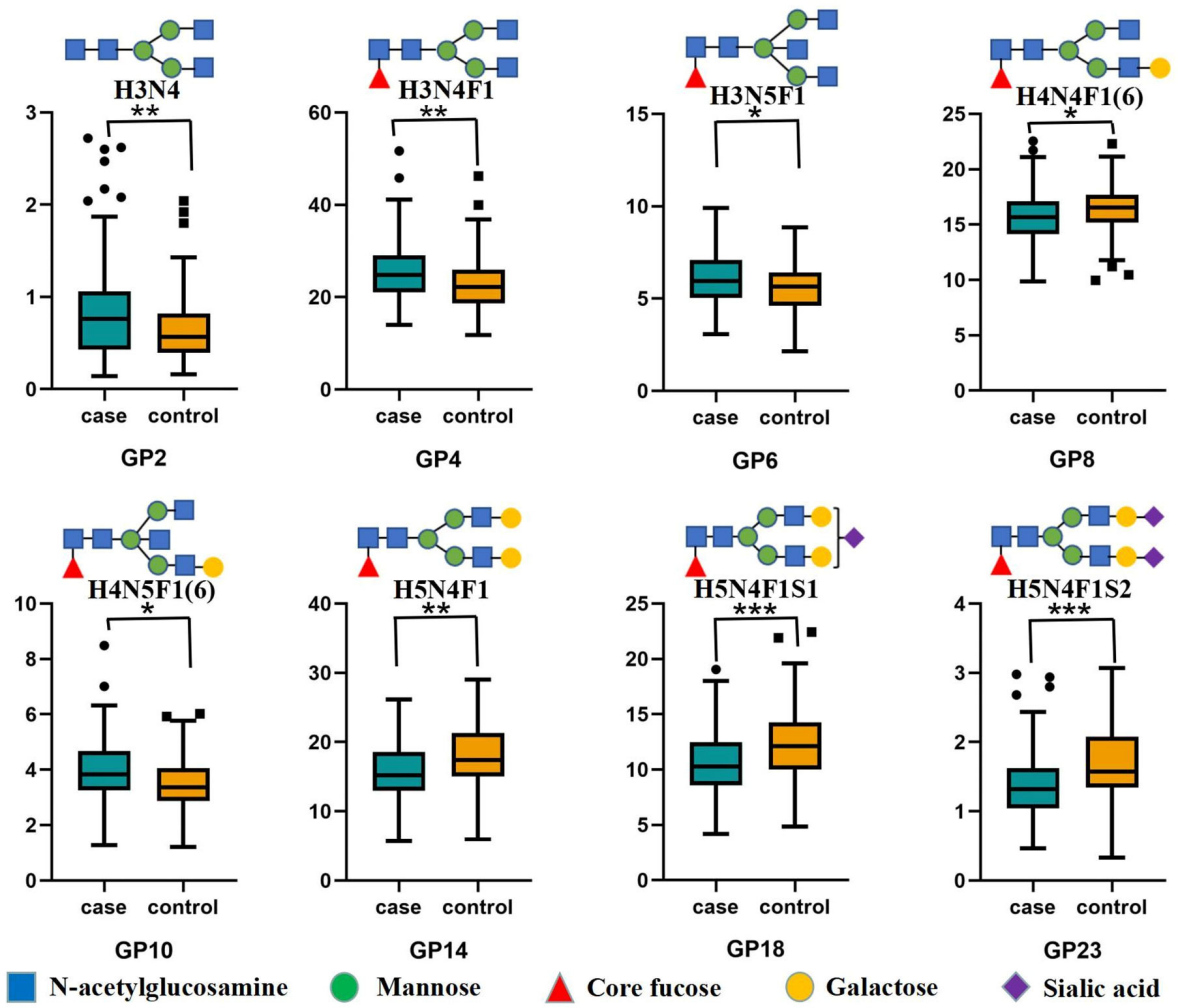
Differences in IgG initial glycans between the LN group and non-LN group Statistically significant, *P < 0.05; **P < 0.01; ***P < 0.001. IgG N-glycosylation levels of 94 patients with lupus nephritis (LN+) and 94 nonlupus nephritis (LN-) patients were analyzed using hydrophilic interaction chromatography (HILIC)-ultra-performance liquid chromatography (UPLC). Significant differences between LN+ and LN- patients in the directly measured glycan structures were observed. Data are shown as box plots. Each box represents the 25th to 75th percentile. The lines inside the box indicate the median. The lines outside the box indicate the 10th and 90th percentiles. Circles indicate outliers. GP, glycan peak; IgG, immunoglobulin G; LN, lupus nephritis.

The median GP10 level was 3.83 (IQR = 3.27 to 4.68) in the LN group, which was significantly higher than that in the control group (M = 3.37, IQR = 2.87 to 4.07). The level of GP8 containing a galactose in the LN group was 15.66 (IQR = 14.14 to 17.09), which was significantly lower than that in the control group (M = 16.55, IQR = 15.17 to 17.68). The level of GP14 with 2 galactoses decreased significantly when compared to the controls (**Table S3**; **Figures 1**, **S2**). Then, the calculated aGal/Gal ratio, G0 and G2 in the derivatized glycans showed statistically significant differences, and the aGal/Gal ratio and the content of G0 in the LN group were greater than those in the control group; in contrast, the content of G2 in the LN group was less than that in the control group (**Table S4**).

### Sialylation of IgG

In the LN group, we observed a statistically significant decrease in all sialylated glycans (Stotal) and in derived trait S1 (**Table S4**). The level of GP18 in the LN group was 10.29 (IQR = 8.58 to 12.48), which was significantly lower than that in the control group (M = 12.11, IQR= 10.02 to 14.25). The level of GP23 with 2 sialic acids decreased significantly compared with the control group (**Table S3**; **Figures 1**, **S2**).

### Core fucosylation and bisecting GlcNAc

The differences in glycans that contained core fucosylation were as follows. The levels of GP4, GP6 and GP10 in the LN group were significantly higher than those in the control group. In contrast, the levels of GP8, GP14, GP18 and GP23 in the LN group were significantly lower than those in the control group (**Table S3**; **Figure 1**). The decrease was expressed in derived traits related to core fucosylation (F, FS, FG1 and FG2). The derivatized glycan FG0 showed a statistically significant increase compared with the control group (**Table S4**). At the same time, major glycans that contained bisecting GlcNAc (GP6 and GP10) increased significantly, and the increase was more pronounced in derived traits (B, BN and BS) that measured bisecting GlcNAc between patients and controls (**Tables S3**, **S4**; **Figures 1**, **S2**).

### Association of IgG N-glycans with the presence of lupus nephritis

**Figure 2:**
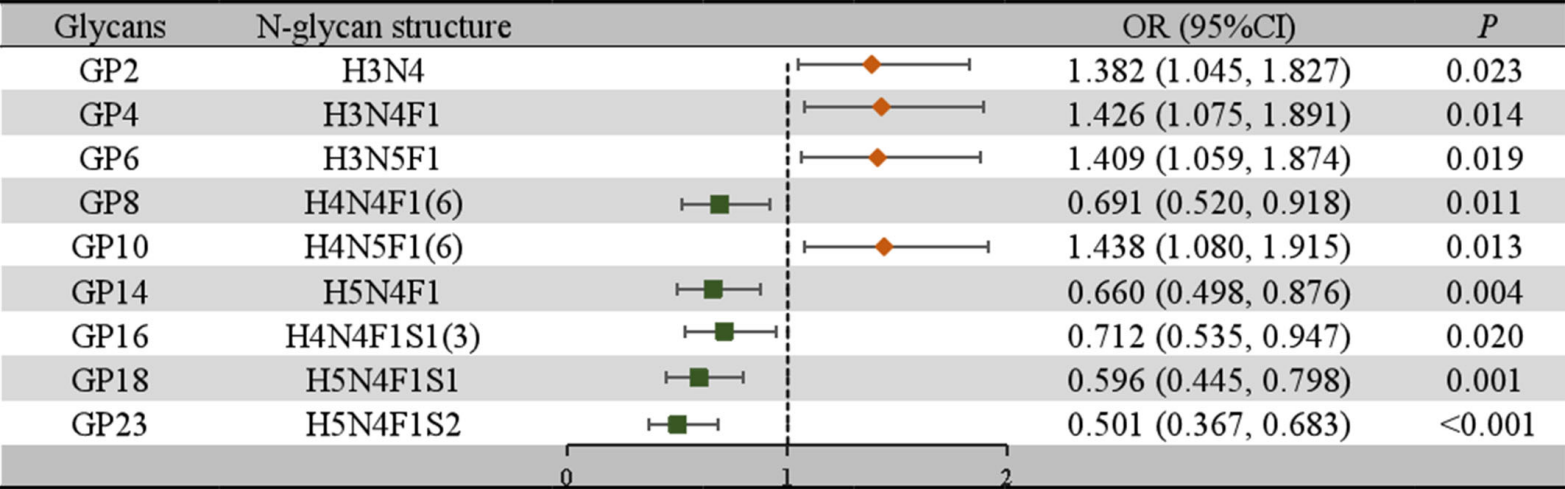
Associations of the normalized initial glycans and LN as determined by multivariate logistic regression analyses. *P* < 0.05 was considered statistically significant using logistic regression analysis. Multivariate logistic regression analyses were performed after adjusting for age, BMI, hypertension, diabetes mellitus, and hyperlipidemia. BMI, body mass index; CI, confidence interval; GP, glycan peak; LN, lupus nephritis; OR, odds ratio.

**Figure 3:**
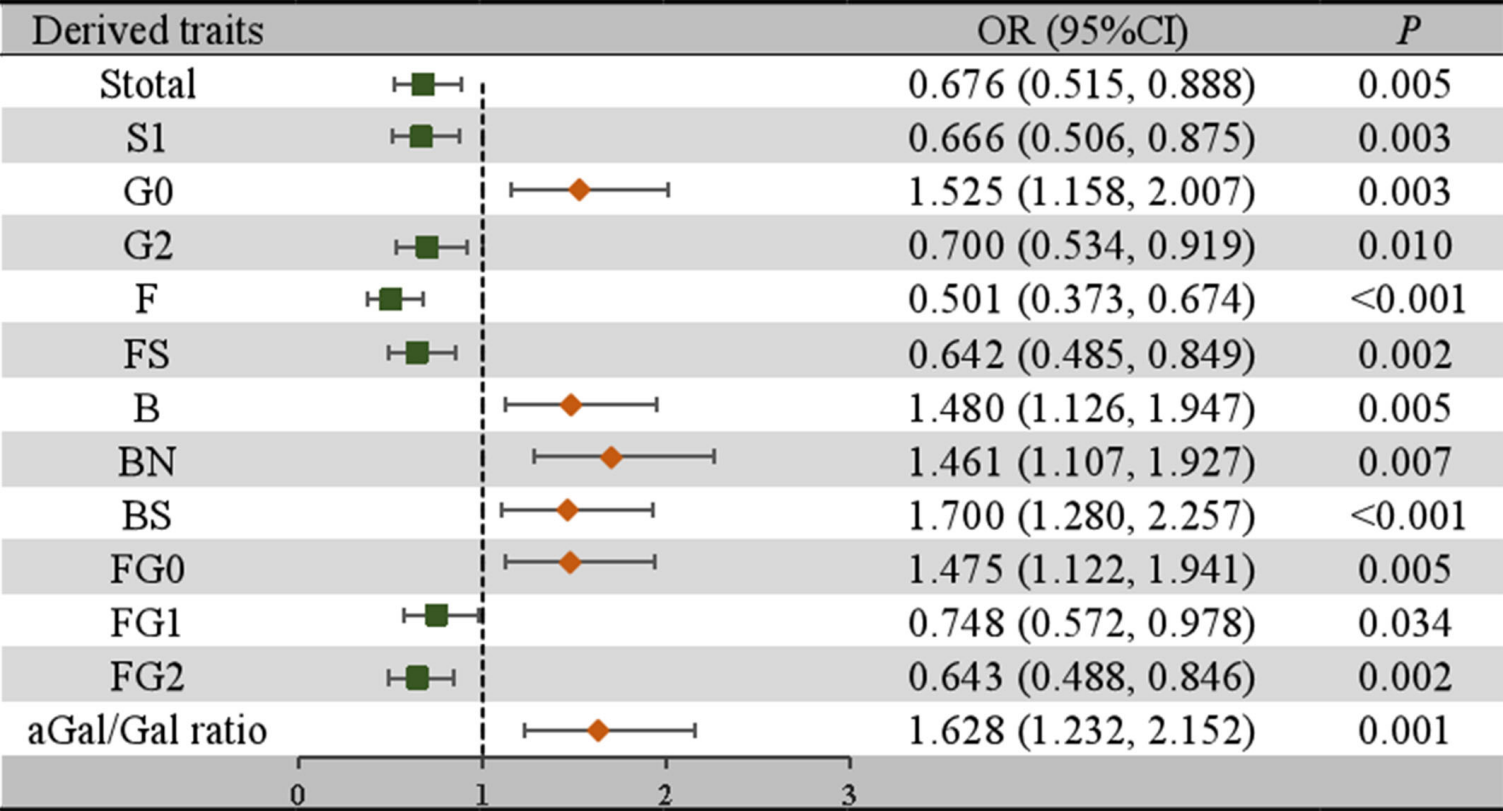
Associations of the derived traits and LN as determined by multivariate logistic regression analyses. *P* < 0.05 was considered statistically significant using logistic regression analysis. Multivariate logistic regression analyses were performed after adjusting for age, BMI, hypertension, diabetes mellitus, and hyperlipidemia. B, bisecting GlcNAc; BMI, body mass index; CI, confidence interval; F, core fucose; G, galactose; GP, glycan peak; LN, lupus nephritis; N, neutral glycans; OR, odds ratio; S, sialic acid.

To further confirm the association between the initial glycans and LN, a multivariable logistic regression analysis was performed. As shown in **Figure 2**, after adjusting for age, BMI, hypertension, diabetes and dyslipidemia, the relative abundance of GP2, GP4, GP6, and GP10 of LN among SLE women were higher, while the relative abundance of GP8, GP14, GP16, GP18, and GP23 were reduced. In addition, the relative abundance of sialic acid glycans (Stotal, OR = 0.676, 95% CI: 0.515-0.888; S1, OR = 0.666, 95% CI: 0.506-0.875) was decreased in the LN group. In the LN group, the relative abundance of galactosylated glycans (G2, OR = 0.700, 95% CI: 0.534-0.919; FG1, OR = 0.748, 95% CI: 0.572-0.978; FG2, OR = 0.643, 95% CI: 0.488-0.846) was decreased, while the relative abundance of nongalactosylated glycans (G0, OR = 1.525, 95% CI: 1.158-2.007; FG0, OR = 1.475, 95% CI: 1.122-1.941, aGal/Gal ratio, OR = 1.628, 95% CI: 1.232-2.152) was increased. The relative abundance of fucosylated glycans (F, OR = 0.501, 95% CI: 0.373-0.674; FS, OR = 0.642, 95% CI: 0.485-0.849) was decreased, and the relative abundance of bisected GlcNAc glycans (B, OR = 1.480, 95% CI: 1.126-1.947; BN, OR = 1.461, 95% CI: 1.107-1.927; BS, OR = 1.700, 95% CI: 1.280-2.257) was increased (**Figure 3**).

### Identification of lupus nephritis using IgG N-glycan markers

**Figure 4:**
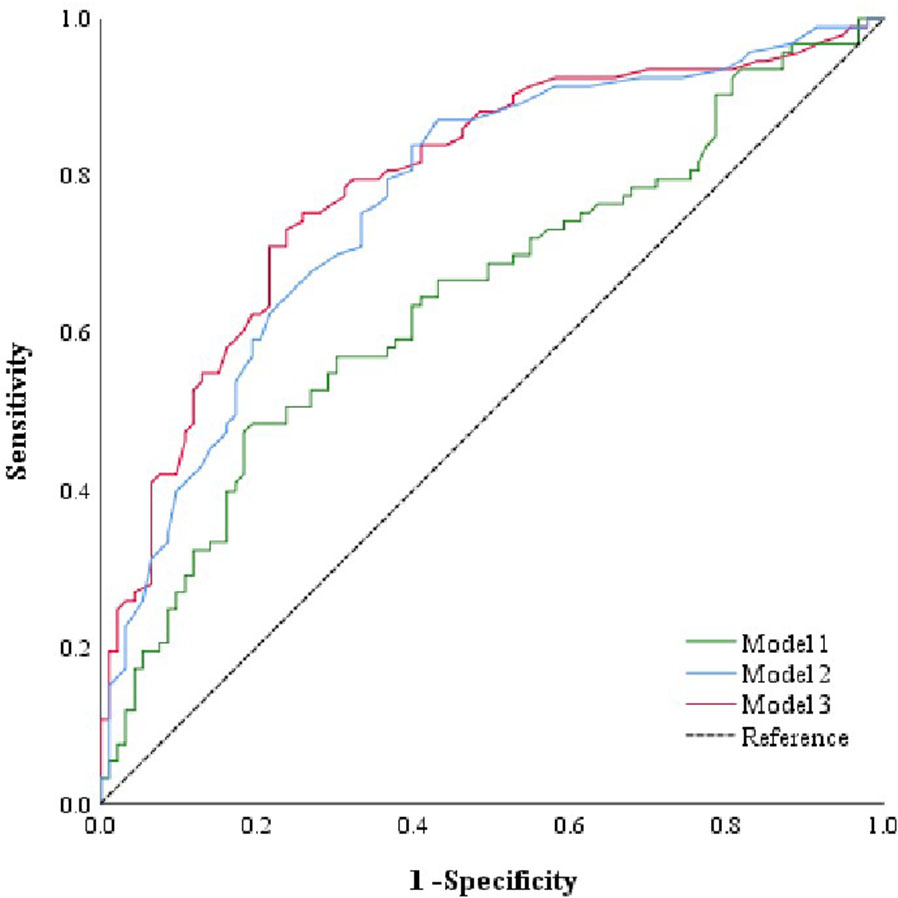
ROC curve analysis of the binary logistic regression model for the prediction of LN. Model 1 consists of GP8, GP10, and GP18. Model 2 consists of anemia and WBC count. Model 3 consists of anemia, GP8, GP10, and GP18. GP, glycan peak; LN, lupus nephritis; ROC, receiver operator characteristic, WBC, white blood cell.

After adjusting for confounding factors, 9 initial glycans (GP2, GP4, GP6, GP8, GP10, GP14, GP16, GP18, and GP23) showed a significant association with LN (**Figure 2**). An internal correlation analysis was conducted among these 9 initial glycans, by which significant correlations were observed (*P* < 0.05) (**Figure S3**). To address multicollinearity issues, a stepwise logistic regression model was used for variable selection, and three diagnostic models were established: 1) Model 1: GP8, GP10, and GP18 were significant and were selected to develop a identification model for LN; 2) Model 2: anemia and the WBC count, which showed significant differences, were included in the identification model; 3) Model 3: GP8, GP10, GP18, and anemia were included in the identification model (**Table S5**). We then employed a multiple-factor logistic regression to establish an LN diagnostic model based on the two indicators, uCRE and eGFR: 4) Model 4: GP8, GP10, GP18, uCRE and eGFR; and 5) Model 5: uCRE and eGFR (**Table S6**). ROC curves were plotted to evaluate the determination performance of the five models (**Figures 4**, **S4**). The discriminatory performance, sensitivity, and specificity of the five models in distinguishing between the case group and control group are shown in **Table S7**. The AUC value of Model 3 was 0.792 (95% CI: 0.727-0.858), which was significantly higher than those of Model 1 (AUC = 0.647, 95% CI: 0.568-0.726), Model 2 (AUC = 0.769, 95% CI: 0.702-0.837), Model 4 (AUC = 0.761, 95% CI: 0.691-0.830), Model 5 (AUC = 0.514, 95% CI: 0.430-0.597).

## Discussion

This study observed a decreased level of sialylation, galactosylation, and core fucosylation, as well as increased bisecting GlcNAc branching, among LN patients when compared to the control group. There is an association between aberrant IgG-N glycosylation and the presence of LN. Additionally, the determination model using IgG N-glycans (GP8, GP10, and GP18) offers potential to improve non-invasive diagnosis of LN among female SLE patients.

Immune-mediated inflammation is the main triggering factor for the development of LN, with mechanisms regulated by both acute and chronic inflammation (37). Inflammatory cytokine alterations in the bodies of SLE patients lead to immune cell abnormalities and renal cell damage. The deposition of autoantibodies or immune complexes exacerbates renal inflammation progression, resulting in the manifestation of LN (38, 39). A study identified various inflammatory mediators associated with LN, including cytokines, chemokines, and glycosaminoglycans, such as interferons (IFNs), IL-6, and TNF-α (40–42). These findings emphasize the significance of inflammatory responses in the occurrence and progression of LN. The abnormal IgG-N glycosylation has the capacity to modulate inflammatory responses through multiple pathways. Moreover, we further discuss potential mechanisms of IgG-N glycosylation in the development of LN.

Sialylation refers to an N-glycosylation modification that is involved in the regulation of the ADCC pathway (43). The reduction in sialylated glycan levels changes the function of IgG, causing a shift from an anti-inflammatory to a proinflammatory state (44–46). Studies have identified decreased levels of sialylated glycans in renal disease patients (47). In another study, a decrease in GP16 and GP18 levels was found in CKD patients (48), aligning with our research findings. Galactosylation, a precursor of sialylation, frequently shows a similar directional alteration (32). Our study revealed a declining trend in both the galactosylation and sialylation of IgG N-glycans. The galactosylation level of the IgG N-glycome is linked to the activation of the lectin pathway of complement activation (49). Decreased terminal galactosylation of IgG N-glycans (50, 51) leads to a reduced binding affinity between antibodies and FcγRIIB, the activation of C1q, the upregulation of the CDC effect, and the promotion of the occurrence and development of inflammation, all of which may contribute to the pathogenesis of LN (52). Previous research indicates that complement system activation occurs in LN patients during disease onset, resulting in immune complex deposition and subsequent renal inflammation and damage (53, 54). Increased levels of agalactosylated glycans are linked to numerous diseases, including idiopathic membranous nephropathy (IMN), where a reduction in the galactosylation of IgG N-glycans has been observed (55). In inflammatory bowel disease, with its proinflammatory potential of IgG, the elevation in agalactosylated glycans in the N-glycome was revealed, which has been reported in multiple inflammatory conditions (56, 57). A study also identified a reduction in the levels of galactosylated glycans and sialylated glycans among female SLE patients, which is consistent with our research findings (49).

The core fucosylated glycans of IgG Fc fragments, through their association with the Fc gamma receptor IIIa (FcgRIIIa) receptor on NK cells, macrophages, and neutrophils, activate the ADCC pathway, thereby upregulating proinflammatory cytokines and triggering an inflammatory response in the body (20, 33). Similar to our findings, a decrease in the level of core fucosylation of IgG N-glycans was discovered in immune thrombocytopenia (ITP) patients, with most patients exhibiting lower levels of core fucosylation being more prone to severe thrombocytopenia (58). Another study also found a significant reduction in the level of core fucosylation of IgG N-glycans in the blood of multiple sclerosis (MS) patients compared to the control group (33). These findings strongly indicate a correlation between the level of core fucosylated glycans and autoimmune diseases. Bisected GlcNAc glycans have been shown to enhance the ADCC pathway mediated by the binding of IgG Fc fragments to the FcγRIII receptor, thus exerting the proinflammatory functions of IgG (59). A study demonstrated that the addition of bisected GlcNAc glycans significantly increased the ADCC effect mediated by noncore fucosylated glycans by several tens of times (60). In a Mendelian randomization study, the causal relationship between the development of SLE and abnormal IgG N-glycosylation modifications was explored, and the findings suggested that the increased risk of SLE is associated with an increase in the relative abundance of N-glycan structures with bisecting GlcNAc in the total IgG N-glycome (61). Elevated bisection of glycans appears to be downstream from autoimmune disease, leading to the conclusion that IgG N-glycosylation traits could serve as biomarkers of SLE (62). All these findings emphasize the regulatory role of IgG N-glycosylation in modulating inflammatory responses in LN patients.

To our knowledge, this study represents the first investigation into the correlation between IgG N-glycans and LN specifically in female SLE patients. These findings offer novel insights into the pathogenesis of LN. However, several limitations must be acknowledged. First, due to the nature of this case-control study, establishing a temporal and causal relationship between IgG N-glycosylation and LN is challenging. Second, the sample size is relatively small, which restricts the generalizability of our findings. Third, we did not classify the degree of LN, such as minimal mesangial LN, mesangial proliferative LN, focal LN, diffuse LN, membranous LN and advanced sclerosing LN, due to the lack of relevant clinical data. And this study did not discuss the effect of creatinine ratio as well as histological parameters due to lack of relevant data. Notwithstanding these limitations, we have conducted an analysis of IgG N-glycosylation profiles in female SLE patients with concurrent LN, contributing to the comprehension of the regulatory role of IgG N-glycosylation in LN development and the identification of potential glycan biomarkers. These findings provide scientific evidence for the early diagnosis of LN. However, further validation in a larger sample of LN patients is necessary. In the future, high-throughput profiling of IgG N-glycans shows promise for the clinical diagnosis of LN.

## Conclusion

This study provides novel insights into the pathogenesis of LN by highlighting a potential association between IgG N-glycans and the observed inflammatory response in SLE patients with concurrent LN. Our findings suggest that alterations in IgG sialylation, galactosylation, core fucosylation and bisecting GlcNAc levels may play important roles in modulating the functional activity of IgG and contribute to the development of LN. Furthermore, IgG N-glycans hold promise as potential biomarkers for differentiating LN patients among female SLE individuals, and the combination of IgG N-glycans with population baseline characteristic indicators enhances the diagnostic efficacy for LN.

## Data availability statement

The original contributions presented in the study are included in the article/**Supplementary Material**. Further inquiries can be directed to the corresponding authors.

## Ethics statement

This study was approved by the Ethics Committee of Shandong First Medical University and conducted according to the guidelines of the Declaration of Helsinki (approval number: 201712). The patients/participants provided their written informed consent to participate in this study.

## Author contributions

XL: Writing – original draft. LW: Writing – original draft. MW: Data curation, Writing – review and editing. YL: Data curation, Writing – review and editing. QZ: Data curation, Writing – review and editing. YS: Formal Analysis, Writing – review and editing. YZ: Formal Analysis, Writing – review and editing. YW: Formal Analysis, Writing – review and editing. WW: Conceptualization, Writing – review and editing. LJ: Writing – review and editing. HH: Writing – review and editing. DL: Writing – review and editing.

## References

[1] Iwamoto T Niewold TB. Genetics of human lupus nephritis. Clin Immunol (2017) 185:32–9. doi: 10.1016/j.clim.2016.09.012 PMC5373939 27693588

[2] Almaani S Meara A Rovin BH. Update on lupus nephritis. Clin J Am Soc Nephrol (2017) 12(5):825–35. doi: 10.2215/CJN.05780616 PMC5477208 27821390

[3] Mahajan A Amelio J Gairy K Kaur G Levy RA Roth D. Systemic lupus erythematosus, lupus nephritis and end-stage renal disease: a pragmatic review mapping disease severity and progression. Lupus (2020) 29(9):1011–20. doi: 10.1177/0961203320932219 PMC7425376 32571142

[4] Ayoub I Wolf BJ Geng L Song H Khatiwada A Tsao BP. Prediction models of treatment response in lupus nephritis. Kidney Int (2022) 101(2):379–89. doi: 10.1016/j.kint.2021.11.014 PMC8792241 34871620

[5] Cheng Y Yang X Zhang X An Z. Analysis of expression levels of IL-17 and IL-34 and influencing factors for prognosis in patients with lupus nephritis. Exp Ther Med (2019) 17(3):2279–83. doi: 10.3892/etm.2019.7168 PMC6364195 30783486

[6] Yap D Chan TM. B cell abnormalities in systemic lupus erythematosus and lupus nephritis-role in pathogenesis and effect of immunosuppressive treatments. Int J Mol Sci (2019) 20(24):6231. doi: 10.3390/ijms20246231 31835612 PMC6940927

[7] Chang A Clark MR Ko K. Cellular aspects of the pathogenesis of lupus nephritis. Curr Opin Rheumatol (2021) 33(2):197–204. doi: 10.1097/BOR.0000000000000777 33394604 PMC7905798

[8] Bhargava R Li H Tsokos GC. Pathogenesis of lupus nephritis: the contribution of immune and kidney resident cells. Curr Opin Rheumatol (2023) 35(2):107–16. doi: 10.1097/BOR.0000000000000887 35797522

[9] Bhat P Radhakrishnan J. B lymphocytes and lupus nephritis: new insights into pathogenesis and targeted therapies. Kidney Int (2008) 73(3):261–8. doi: 10.1038/sj.ki.5002663 18004299

[10] Yao M Gao C Zhang C Di X Liang W Sun W. Identification of molecular markers associated with the pathophysiology and treatment of lupus nephritis based on integrated transcriptome analysis. Front Genet (2020) 11:583629. doi: 10.3389/fgene.2020.583629 33384713 PMC7770169

[11] Fu Q Wu C Dai M Wang S Xu J Dai L. Leflunomide versus azathioprine for maintenance therapy of lupus nephritis: a prospective, multicentre, randomised trial and long-term follow-up. Ann Rheum Dis (2022) 81(11):1549–55. doi: 10.1136/ard-2022-222486 PMC9606489 35788493

[12] Hudspeth K Wang S Wang J Rahman S Smith MA Casey KA. Natural killer cell expression of Ki67 is associated with elevated serum IL-15, disease activity and nephritis in systemic lupus erythematosus. Clin Exp Immunol (2019) 196(2):226–36. doi: 10.1111/cei.13263 PMC6468178 30693467

[13] Radovani B Vuckovic F Maggioni AP Ferrannini E Lauc G Gudelj I. IgG N-glycosylation is altered in coronary artery disease. Biomolecules (2023) 13(2):375. doi: 10.3390/biom13020375 36830744 PMC9953309

[14] Pan H Wu Z Zhang H Zhang J Liu Y Li Z. Identification and validation of IgG N-glycosylation biomarkers of esophageal carcinoma. Front Immunol (2023) 14:981861. doi: 10.3389/fimmu.2023.981861 36999031 PMC10043232

[15] Singh SS Heijmans R Meulen C Lieverse AG Gornik O Sijbrands E. Association of the IgG N-glycome with the course of kidney function in type 2 diabetes. BMJ Open Diabetes Res Care (2020) 8(1):e001026. doi: 10.1136/bmjdrc-2019-001026 PMC7213753 32349995

[16] Lauc G Huffman JE Pucic M Zgaga L Adamczyk B Muzinic A. Loci associated with N-glycosylation of human immunoglobulin G show pleiotropy with autoimmune diseases and haematological cancers. PloS Genet (2013) 9(1):e1003225. doi: 10.1371/journal.pgen.1003225 23382691 PMC3561084

[17] Zhou X Motta F Selmi C Ridgway WM Gershwin ME Zhang W. Antibody glycosylation in autoimmune diseases. Autoimmun Rev (2021) 20(5):102804. doi: 10.1016/j.autrev.2021.102804 33727152 PMC8058319

[18] Liu P Wang X Dun A Li Y Li H Wang L. High-throughput profiling of serological immunoglobulin g n-glycome as a noninvasive biomarker of gastrointestinal cancers. Engineering (2023). doi: 10.1016/j.eng.2023.02.008

[19] Hou H Yang H Liu P Huang C Wang M Li Y. Profile of immunoglobulin G N-glycome in COVID-19 patients: A case-control study. Front Immunol (2021) 12:748566. doi: 10.3389/fimmu.2021.748566 34630427 PMC8495247

[20] Li Y Shi F Wang G Lv J Zhang H Jin H. Expression profile of immunoglobulin G glycosylation in children with epilepsy in han nationality. Front Mol Neurosci (2022) 15:843897. doi: 10.3389/fnmol.2022.843897 35845609 PMC9283856

[21] Varadi C Hajdu V Farkas F Gilanyi I Olah C Viskolcz B. The analysis of human serum N-glycosylation in patients with primary and metastatic brain tumors. Life (Basel) (2021) 11(1):29. doi: 10.3390/life11010029 33418875 PMC7825111

[22] Haslund-Gourley BS Wigdahl B Comunale MA. IgG N-glycan signatures as potential diagnostic and prognostic biomarkers. Diagn (Basel) (2023) 13(6):1016. doi: 10.3390/diagnostics13061016 PMC10047871 36980324

[23] Gyebrovszki B Acs A Szabo D Auer F Novozanszki S Rojkovich B. The role of igG fc region N-glycosylation in the pathomechanism of rheumatoid arthritis. Int J Mol Sci (2022) 23(10):5828. doi: 10.3390/ijms23105828 35628640 PMC9146365

[24] Siekman SL Pongracz T Wang W Nouta J Kremsner PG Da SP. The IgG glycome of SARS-CoV-2 infected individuals reflects disease course and severity. Front Immunol (2022) 13:993354. doi: 10.3389/fimmu.2022.993354 36389824 PMC9641981

[25] Stambuk J Vuckovic F Habazin S Hanic M Novokmet M Nikolaus S. Distinct longitudinal changes in immunoglobulin G N-glycosylation associate with therapy response in chronic inflammatory diseases. Int J Mol Sci (2022) 23(15). doi: 10.3390/ijms23158473 PMC9368836 35955616

[26] Sjowall C Zapf J von Lohneysen S Magorivska I Biermann M Janko C. Altered glycosylation of complexed native IgG molecules is associated with disease activity of systemic lupus erythematosus. Lupus (2015) 24(6):569–81. doi: 10.1177/0961203314558861 25389233

[27] Zhang W Tang Z Shi Y Ji L Chen X Chen Y. Association between gamma-glutamyl transferase, total bilirubin and systemic lupus erythematosus in Chinese women. Front Immunol (2021) 12:682400. doi: 10.3389/fimmu.2021.682400 34276670 PMC8277571

[28] Aringer M Costenbader K Daikh D Brinks R Mosca M Ramsey-Goldman R. European League Against Rheumatism/American College of Rheumatology classification criteria for systemic lupus erythematosus. Ann Rheum Dis (2019) 78(9):1151–9. doi: 10.1136/annrheumdis-2018-214819 31383717

[29] Zhang H Yang NS Lu J Cao H Du R Zhang W. Recommendations for the diagnosis and management of lupus nephritis in China. Zhonghua Nei Ke Za Zhi (2021) 60(9):784–90. doi: 10.3760/cma.j.cn112138-20210609-00410 34445813

[30] Wang M Chen X Tang Z Zhang W Hou H Sun X. Association between immunoglobulin G N-glycosylation and vascular cognitive impairment in a sample with atherosclerosis: A case-control study. Front Aging Neurosci (2022) 14:823468. doi: 10.3389/fnagi.2022.823468 35221999 PMC8868374

[31] Qin R Yang Y Chen H Qin W Han J Gu Y. Prediction of neoadjuvant chemotherapeutic efficacy in patients with locally advanced gastric cancer by serum IgG glycomics profiling. Clin Proteomics (2020) 17:4. doi: 10.1186/s12014-020-9267-8 32042279 PMC7003487

[32] Theodoratou E Thaçi K Agakov F Timofeeva MN Štambuk J Pučić-Baković M. Glycosylation of plasma IgG in colorectal cancer prognosis. Sci Rep (2016) 6(1):28098. doi: 10.1038/srep28098 27302279 PMC4908421

[33] Cvetko A Kifer D Gornik O Klaric L Visser E Lauc G. Glycosylation alterations in multiple sclerosis show increased proinflammatory potential. Biomedicines (2020) 8(10):410. doi: 10.3390/biomedicines8100410 33065977 PMC7599553

[34] Adua E Memarian E Russell A Trbojevic-Akmacic I Gudelj I Juric J. High throughput profiling of whole plasma N-glycans in type II diabetes mellitus patients and healthy individuals: A perspective from a Ghanaian population. Arch Biochem Biophys (2019) 661:10–21. doi: 10.1016/j.abb.2018.10.015 30365935

[35] Qin R Yang Y Qin W Han J Chen H Zhao J. The value of serum immunoglobulin G glycome in the preoperative discrimination of peritoneal metastasis from advanced gastric cancer. J Cancer (2019) 10(12):2811–21. doi: 10.7150/jca.31380 PMC6584920 31258789

[36] Lv H Yang X Zhou Y Wu J Liu H Wang Y. Parity and serum lipid levels: a cross-sectional study in chinese female adults. Sci Rep (2016) 6:33831. doi: 10.1038/srep33831 27645134 PMC5028753

[37] Qi S Chen Q Xu D Xie N Dai Y. Clinical application of protein biomarkers in lupus erythematosus and lupus nephritis. Lupus (2018) 27(10):1582–90. doi: 10.1177/0961203318773643 29720035

[38] Cervera R Khamashta MA Font J Sebastiani GD Gil A Lavilla P. Morbidity and mortality in systemic lupus erythematosus during a 10-year period: a comparison of early and late manifestations in a cohort of 1,000 patients. Med (Baltimore) (2003) 82(5):299–308. doi: 10.1097/01.md.0000091181.93122.55 14530779

[39] Pons-Estel GJ Serrano R Plasin MA Espinosa G Cervera R. Epidemiology and management of refractory lupus nephritis. Autoimmun Rev (2011) 10(11):655–63. doi: 10.1016/j.autrev.2011.04.032 21565286

[40] Lech M Anders HJ. The pathogenesis of lupus nephritis. J Am Soc Nephrol (2013) 24(9):1357–66. doi: 10.1681/ASN.2013010026 PMC3752952 23929771

[41] Yan JJ Jambaldorj E Lee JG Jang JY Shim JM Han M. Granulocyte colony-stimulating factor treatment ameliorates lupus nephritis through the expansion of regulatory T cells. BMC Nephrol (2016) 17(1):175. doi: 10.1186/s12882-016-0380-x 27846813 PMC5111287

[42] Gottschalk TA Tsantikos E Hibbs ML. Pathogenic inflammation and its therapeutic targeting in systemic lupus erythematosus. Front Immunol (2015) 6:550. doi: 10.3389/fimmu.2015.00550 26579125 PMC4623412

[43] Dall’Olio F Vanhooren V Chen CC Slagboom PE Wuhrer M Franceschi C. N-glycomic biomarkers of biological aging and longevity: a link with inflammaging. Ageing Res Rev (2013) 12(2):685–98. doi: 10.1016/j.arr.2012.02.002 22353383

[44] Zhang X Yuan H Lyu J Meng X Tian Q Li Y. Association of dementia with immunoglobulin G N-glycans in a Chinese Han Population. NPJ Aging Mech Dis (2021) 7(1):3. doi: 10.1038/s41514-021-00055-w 33542243 PMC7862610

[45] Russell AC Simurina M Garcia MT Novokmet M Wang Y Rudan I. The N-glycosylation of immunoglobulin G as a novel biomarker of Parkinson’s disease. Glycobiology (2017) 27(5):501–10. doi: 10.1093/glycob/cwx022 28334832

[46] Bhargava R Lehoux S Maeda K Tsokos MG Krishfield S Ellezian L. Aberrantly glycosylated IgG elicits pathogenic signaling in podocytes and signifies lupus nephritis. JCI Insight (2021) 6(9):e147789. doi: 10.1172/jci.insight.147789 33784256 PMC8262331

[47] Barrios C Zierer J Gudelj I Stambuk J Ugrina I Rodriguez E. Glycosylation profile of igG in moderate kidney dysfunction. J Am Soc Nephrol (2016) 27(3):933–41. doi: 10.1681/ASN.2015010109 PMC4769202 26185202

[48] Kao CC Wang SY Chuang YK Lee WY Chang WC Wu MS. Clinical mass spectrometry discovered human igG sialylation as a potential biosignature for kidney function. J Pers Med (2021) 11(8):761. doi: 10.3390/jpm11080761 34442405 PMC8401842

[49] Vuckovic F Kristic J Gudelj I Teruel M Keser T Pezer M. Association of systemic lupus erythematosus with decreased immunosuppressive potential of the IgG glycome. Arthritis Rheumatol (2015) 67(11):2978–89. doi: 10.1002/art.39273 PMC4626261 26200652

[50] Karsten CM Pandey MK Figge J Kilchenstein R Taylor PR Rosas M. Anti-inflammatory activity of IgG1 mediated by Fc galactosylation and association of FcgammaRIIB and dectin-1. Nat Med (2012) 18(9):1401–6. doi: 10.1038/nm.2862 PMC3492054 22922409

[51] Gornik O Pavic T Lauc G. Alternative glycosylation modulates function of IgG and other proteins - implications on evolution and disease. Biochim Biophys Acta (2012) 1820(9):1318–26. doi: 10.1016/j.bbagen.2011.12.004 22183029

[52] Wang J Huang C Zhou J Zhao K Li Y. Causal link between immunoglobulin G glycosylation and cancer: A potential glycobiomarker for early tumor detection. Cell Immunol (2021) 361:104282. doi: 10.1016/j.cellimm.2021.104282 33453507

[53] Thurman JM Yapa R. Complement therapeutics in autoimmune disease. Front Immunol (2019) 10:672. doi: 10.3389/fimmu.2019.00672 31001274 PMC6456694

[54] Park MH Caselman N Ulmer S Weitz IC. Complement-mediated thrombotic microangiopathy associated with lupus nephritis. Blood Adv (2018) 2(16):2090–4. doi: 10.1182/bloodadvances.2018019596 PMC6113612 30131343

[55] Chinello C de Haan N Capitoli G Trezzi B Radice A Pagani L. Definition of igG subclass-specific glycopatterns in idiopathic membranous nephropathy: aberrant igG glycoforms in blood. Int J Mol Sci (2022) 23(9):4664. doi: 10.3390/ijms23094664 35563055 PMC9101794

[56] Gornik O Lauc G. Glycosylation of serum proteins in inflammatory diseases. Dis Markers (2008) 25(4-5):267–78. doi: 10.1155/2008/493289 PMC3827815 19126970

[57] Trbojevic AI Ventham NT Theodoratou E Vuckovic F Kennedy NA Kristic J. Inflammatory bowel disease associates with proinflammatory potential of the immunoglobulin G glycome. Inflammation Bowel Dis (2015) 21(6):1237–47. doi: 10.1097/MIB.0000000000000372 PMC4450892 25895110

[58] Wang W Xu X Huang C Gao C. N-glycan profiling alterations of serum and immunoglobulin G in immune thrombocytopenia. J Clin Lab Anal (2022) 36(2):e24201. doi: 10.1002/jcla.24201 34957618 PMC8842136

[59] Menni C Gudelj I Macdonald-Dunlop E Mangino M Zierer J Besic E. Glycosylation profile of immunoglobulin G is cross-sectionally associated with cardiovascular disease risk score and subclinical atherosclerosis in two independent cohorts. Circ Res (2018) 122(11):1555–64. doi: 10.1161/CIRCRESAHA.117.312174 PMC5970566 29535164

[60] Yi CH Ruan CP Wang H Xu XY Zhao YP Fang M. Function characterization of a glyco-engineered anti-EGFR monoclonal antibody cetuximab in *vitro* . Acta Pharmacol Sin (2014) 35(11):1439–46. doi: 10.1038/aps.2014.77 PMC4220073 25263334

[61] Zaytseva OO Sharapov SZ Perola M Esko T Landini A Hayward C. Investigation of the causal relationships between human IgG N-glycosylation and 12 common diseases associated with changes in the IgG N-glycome. Hum Mol Genet (2022) 31(10):1545–59. doi: 10.1093/hmg/ddab335 34791244

[62] Zou G Ochiai H Huang W Yang Q Li C Wang LX. Chemoenzymatic synthesis and Fcgamma receptor binding of homogeneous glycoforms of antibody Fc domain. Presence of a bisecting sugar moiety enhances the affinity of Fc to FcgammaIIIa receptor. J Am Chem Soc (2011) 133(46):18975–91. doi: 10.1021/ja208390n PMC3218234 22004528

