# Supplementary material for: Association between immunoglobulin G N-glycosylation and lupus nephritis in female patients with systemic lupus erythematosus: a case-control study
Source: Front Immunol. 2023 Sep 21;14:1257906. doi: 10.3389/fimmu.2023.1257906 (PMC10552529; doi:10.3389/fimmu.2023.1257906)
Supplement: Supplementary file 1 [file DataSheet_1.docx]

**Supplementary**


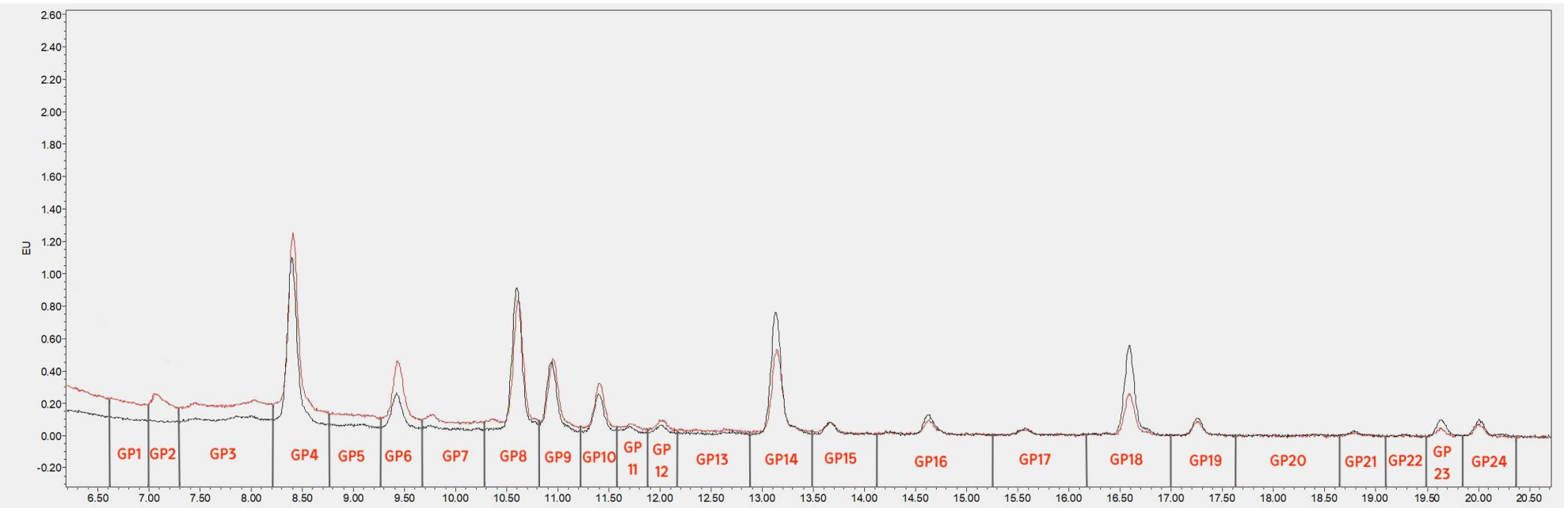


Figure S1. A chromatogram showing differential N-glycan peaks of an individual with or without LN. *There is a statistical difference between the case group and the control group (P<0.05). Red represents the distribution of N-glycan peaks of a case; black represents the distribution of N-glycan peaks of a control. This figure shows the difference in initial glycans between individuals with or without LN, and it cannot fully reflect all the differences between the initial glycans in the overall case group and the control group. GP, glycan peak; LN, lupus nephritis.


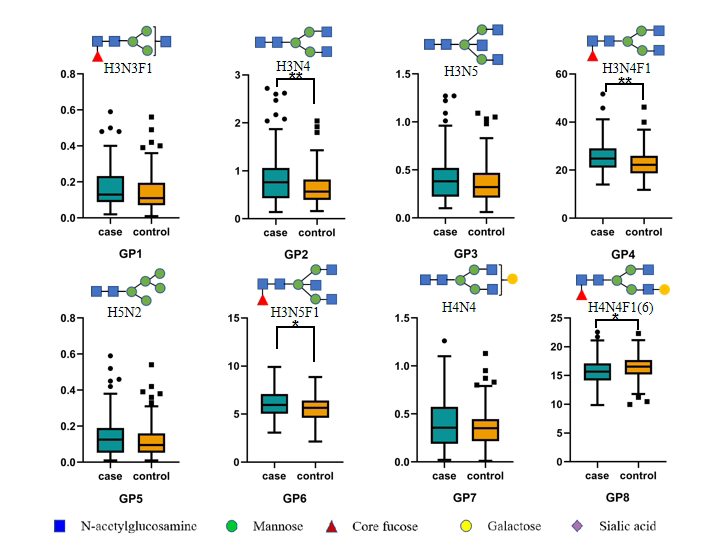


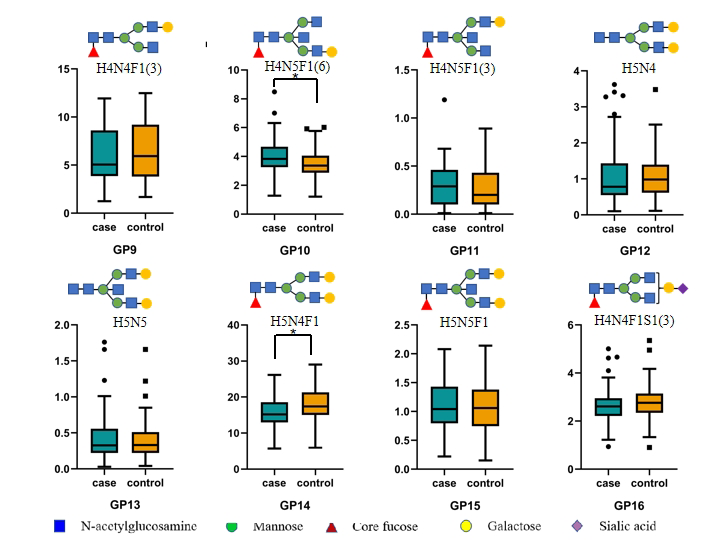


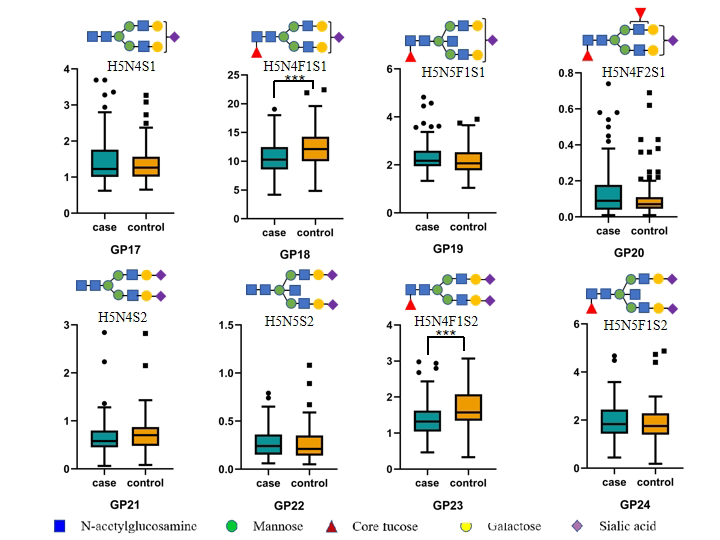


Figure S2. Structures of the initial IgG glycome. Blue squares represent bisecting GlcNAc; green circles represent mannose; red triangles represent fucose; yellow circles represent galactose; purple diamonds represent sialic acid. GP, glycan peak; GlcNAc, N-acetylglucosamine; IgG, immunoglobulin G.


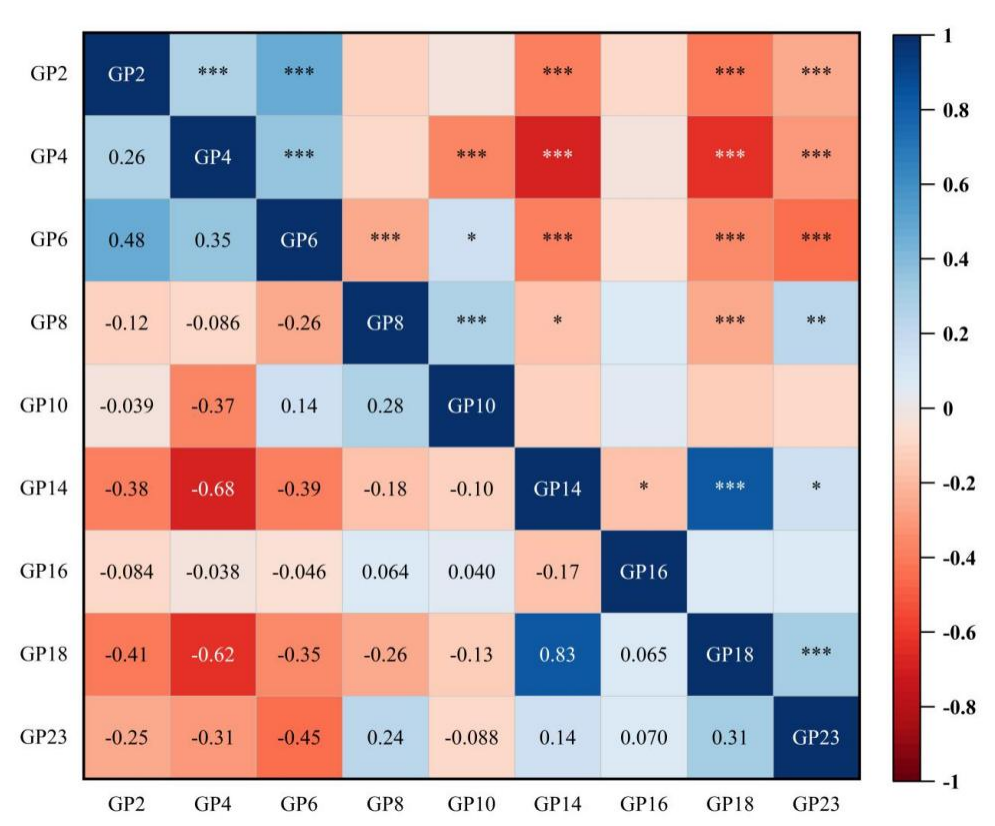


Figure S3. The correlation coefficient of independent glycans associated with LN obtained by Spearman correlation analysis. Statistically significant, **P* < 0.05; ***P* < 0.01; ****P* < 0.001. *P* values were calculated by Spearman correlation analysis. The positive correlations are represented by red, while negative correlations are represented by blue. GP, glycan peak; LN, Lupus nephritis.


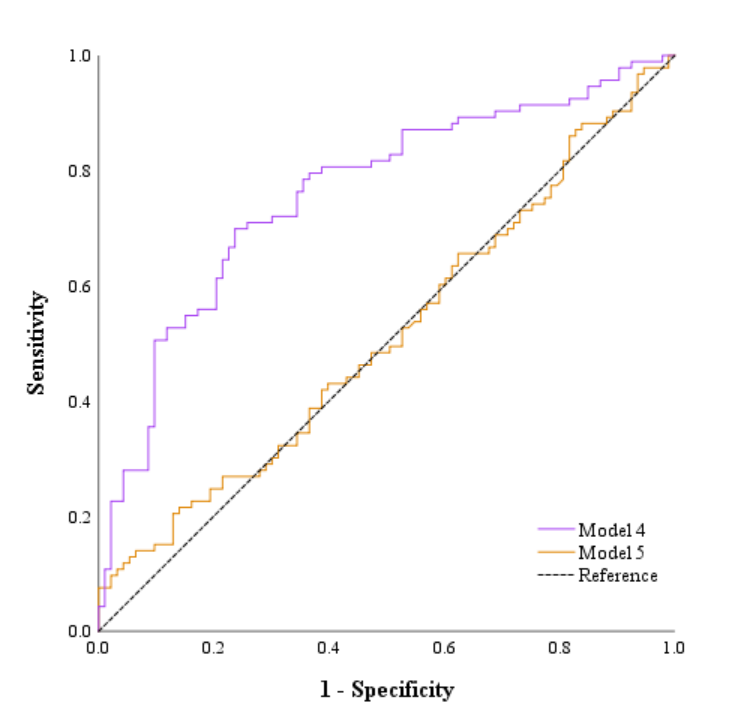


FIGURE S4. ROC curve analysis of the binary logistic regression model for the prediction of LN. Model 4 consists of Scr, eGFR, GP8, GP10, and GP18. Model 5 consists of Scr and eGFR. GP, glycan peak; eGFR, estimated glomerular filtration rate; LN, lupus nephritis; ROC, receiver operator characteristic; Scr, Serum creatinine.

Table S1 The calculation formula of Initial glycans

| Initial glycans | Glycan composition | Glycan structure | Observed m/z[M+H]+ | Formula |
| --- | --- | --- | --- | --- |
| GP1 | H3N3F1 | FA1 | 1380.5101 | GP1/GP*100 |
| GP2 | H3N4 | A2 | 1437.5302 | GP2/GP*100 |
| GP3 | H3N5 | A2B | 1640.6062 | GP3/GP*100 |
| GP4 | H3N4F1 | FA2 | 1583.5818 | GP4/GP*100 |
| GP5 | H5N2 | M5 | 1355.4761 | GP5/GP*100 |
| GP6 | H3N5F1 | FA2B | 1786.699 | GP6/GP*100 |
| GP7 | H4N4 | A2[3]G1 | 1599.5766 | GP7/GP*100 |
| GP8 | H4N4F1(6) | FA2[6]G1 | 1745.6307 | GP8/GP*100 |
| GP9 | H4N4F1(3) | FA2[3]G1 | 1745.6276 | GP9/GP*100 |
| GP10 | H4N5F1(6) | FA2[6]BG1 | 1948.7034 | GP10/GP*100 |
| GP11 | H4N5F1(3) | FA2[3]BG1 | 1948.7022 | GP11/GP*100 |
| GP12 | H5N4 | A2G2 | 1761.6235 | GP12/GP*100 |
| GP13 | H5N5 | A2BG2 | 1964.6982 | GP13/GP*100 |
| GP14 | H5N4F1 | FA2G2 | 1907.6764 | GP14/GP*100 |
| GP15 | H5N5F1 | FA2BG2 | 2110.7544 | GP15/GP*100 |
| GP16 | H4N4F1S1(3) | FA2G1S1 | 2036.7205 | GP16/GP*100 |
| GP17 | H5N4S1 | A2G2S1 | 2052.7108 | GP17/GP*100 |
| GP18 | H5N4F1S1 | FA2G2S1 | 2198.8215 | GP18/GP*100 |
| GP19 | H5N5F1S1 | FA2BG2S1 | 2401.839 | GP19/GP*100 |
| GP20 | H5N4F2S1 | FA2FG2S1 | 2344.905 | GP20/GP*100 |
| GP21 | H5N4S2 | A2G2S2 | 2343.8028 | GP21/GP*100 |
| GP22 | H5N5S2 | A2BG2S2 | 2546.8809 | GP22/GP*100 |
| GP23 | H5N4F1S2 | FA2G2S2 | 2489.8596 | GP23/GP*100 |
| GP24 | H5N5F1S2 | FA2BG2S2 | 2692.9315 | GP24/GP*100 |

composition abbreviations-Hx, number (x) of hexose residues; Nx, number (x) of GlcNAc residues; Fx, number (x) of fucose residues; Sx, number (x) of N-acetylneuraminic acid residues.structure abbreviations-all N-glycans have two core GlcNAcs; F at the start of the abbreviation indicates a core-fucose α 1,6-linked to the inner GlcNAc; Mx, number (x) of mannose on core GlcNAcs; Ax, number of antenna (GlcNAc) on trimannosyl core; A2, biantennary with both GlcNAcs as β 1,2-linked; B, bisecting GlcNAc linked β1,4 to β 1,3 mannose; G (x), number (x) of β 1,4 linked galactose on antenna; F (x), number (x) of fucose linked α 1,3 to antenna GlcNAc; S (x), number (x) of sialic acids linked to galactoses.

Table S2 The calculation formula of derived traits

| **Derived traits** | **Description** | **Computational method** |
| --- | --- | --- |
| GPN | Proportion of neutral glycans in total IgG glycans | GP1+GP2+GP3+GP4+GP5+GP6+GP7+GP8+GP9+GP10+GP11+GP12+GP13+GP14+GP15 |
| Stotal | Proportion of sialylated glycans in total IgG glycans | GP16+GP17+GP18+GP19+GP21+GP22+GP23+GP24 |
| S1 | Proportion of monosialylated glycans in total IgG glycans | GP16+GP17+GP18+GP19 |
| S2 | Proportion of disialylated glycans in total IgG glycans | GP21+GP22+GP23+GP24 |
| G0 | Proportion of agalactosylated glycans in total IgG glycans | GP1+ GP2+ GP3+ GP4+ GP6 |
| G1 | Proportion of monogalactosylated glycans in total IgG glycans | GP7+ GP8+ GP9+ GP10+ GP11 |
| G2 | Proportion of galactosylated glycans in total IgG glycans | GP12+ GP13+ GP14+ GP15 |
| F | Proportion of fucosylated glycans in total IgG glycans | GP1+ GP4+ GP6+ GP8+ GP9+ GP10+ GP11+ GP14+ GP15+ GP16+ GP18+ GP19+ GP23+ GP24 |
| FN | Proportion of fucosylated glycans in total neutral IgG glycans | (GP1+ GP4+ GP6+ GP8+ GP9+ GP10+ GP11+ GP14+ GP15)/GPN*100 |
| FS | Proportion of fucosylated glycans in total sialylated IgG glycans | (GP16+ GP18+ GP19+ GP23+ GP24)/Stotal*100 |
| B | Proportion of bisecting glycans in total IgG glycans | GP3+ GP6+ GP10+ GP11+ GP13+ GP15+ GP19+ GP22+ GP24 |
| BN | Proportion of bisecting glycans in neutral IgG glycans | (GP3+ GP6+ GP10+ GP11+ GP13)/GPN*100 |
| BS | Proportion of bisecting glycans in sialylated IgG glycans | (GP19+ GP22+ GP24)/Stotal*100 |
| FG0 | Proportion of fucosylated agalactosylated glycans in total IgG glycans | GP4 |
| FG1 | Proportion of fucosylated monogalactosylated glycans in total IgG glycans | GP8 + GP9 |
| FG2 | Proportion of fucosylated galactosylated glycans in total IgG glycans | GP14 |
| aGal/Gal ratio | the relative intensities of agalactosylated (G0) vs monogalactosyl (G1) and digalactosyl (G2) N-glycans | G0/(G1 + G2*2)*100 |

B, bisecting GlcNAc; F , core fucose; G, galactose; N, neutral glycans; S, sialic acid.

Table S3. Levels of initial glycans in the LN group and non-LN group

| Initial glycans | LN group  (n=94) | Non-LN group (n=94) | Z | P |
| --- | --- | --- | --- | --- |
|  |  |  |  |  |
| GP1 | 0.13 (0.09, 0.25) | 0.11 (0.07, 0.20) | 1.936 | 0.053 |
| GP2 | 0.76 (0.44, 1.11) | 0.57 (0.40, 0.82) | 2.609 | 0.009* |
| GP3 | 0.38 (0.23, 0.55) | 0.32 (0.21, 0.47) | 1.357 | 0.175 |
| GP4 | 24.78 (21.05, 29.10) | 22.23 (18.69, 25.94) | 2.954 | 0.003* |
| GP5 | 0.13 (0.06, 0.20) | 0.10 (0.06, 0.17) | 1.083 | 0.279 |
| GP6 | 5.95 (5.05, 7.09) | 5.66 (4.62, 6.42) | 2.168 | 0.030* |
| GP7 | 0.36 (0.19, 0.59) | 0.35 (0.22, 0.45) | 0.906 | 0.365 |
| GP8 | 15.66 (14.14, 17.09) | 16.55 (15.17, 17.68) | 2.563 | 0.010* |
| GP9 | 5.06 (3.86, 8.61) | 5.93 (3.81, 9.21) | 0.928 | 0.353 |
| GP10 | 3.83 (3.27, 4.68) | 3.37 (2.87, 4.07) | 2.169 | 0.030* |
| GP11 | 0.29 (0.10, 0.46) | 0.20 (0.10, 0.43) | 1.261 | 0.207 |
| GP12 | 0.80 (0.56, 1.46) | 0.98 (0.62, 1.40) | 0.461 | 0.645 |
| GP13 | 0.33 (0.22, 0.58) | 0.33 (0.22, 0.51) | 0.314 | 0.754 |
| GP14 | 15.20 (12.99, 18.56) | 17.43 (15.04, 21.27) | 3.352 | 0.001* |
| GP15 | 1.04 (0.80, 1.43) | 1.06 (0.75, 1.38) | 0.457 | 0.648 |
| GP16 | 2.61 (2.21, 2.95) | 2.76 (2.35, 3.15) | 1.771 | 0.077 |
| GP17 | 1.25 (1.01, 1.82) | 1.26 (1.01, 1.57) | 0.273 | 0.785 |
| GP18 | 10.29 (8.58, 12.48) | 12.11 (10.02, 14.25) | 4.137 | <0.001* |
| GP19 | 2.18 (1.94, 2.62) | 2.07 (1.77, 2.53) | 1.388 | 0.165 |
| GP20 | 0.09 (0.04, 0.19) | 0.07 (0.05, 0.11) | 1.342 | 0.18 |
| GP21 | 0.58 (0.46, 0.84) | 0.70 (0.49, 0.89) | 1.251 | 0.211 |
| GP22 | 0.24 (0.15, 0.38) | 0.21 (0.14, 0.36) | 0.73 | 0.465 |
| GP23 | 1.32 (1.05, 1.62) | 1.57 (1.35, 2.08) | 3.96 | <0.001* |
| GP24 | 1.83 (1.43, 2.44) | 1.75 (1.39, 2.28) | 0.716 | 0.474 |

*Statistically significant, *P* < 0.05. *P* values were calculated by the Mann‒Whitney U test. GP, glycan peak; LN, lupus nephritis.

Table S4. Comparison of IgG N-glycan-derived traits between the LN group and the non-LN group

| Derived traits | LN group (n=94) | Non-LN group (n=94) | *Z* | *P* |
| --- | --- | --- | --- | --- |
| GPN | 75.71 (73.23, 78.32) | 74.65 (71.00, 77.84) | 1.215 | 0.224 |
| Stotal | 18.80 (16.28, 21.47) | 20.39 (17.71, 23.31) | 3.077 | 0.002* |
| S1 | 14.86 (13.14, 17.00) | 16.33 (14.51, 18.61) | 3.225 | 0.001* |
| S2 | 3.62 (2.89, 4.34) | 3.65 (2.98, 4.55) | 1.077 | 0.281 |
| G0 | 27.78 (25.33, 31.94) | 25.03 (21.65, 29.59) | 3.364 | 0.001* |
| G1 | 21.98 (19.57, 24.95) | 23.60 (20.07, 26.13) | 1.094 | 0.274 |
| G2 | 15.38 (11.86, 18.30) | 17.21 (14.92, 20.12) | 2.934 | 0.003* |
| F | 92.43 (89.29, 94.34) | 94.94 (92.66, 95.84) | 4.828 | <0.001* |
| FN | 94.24 (91.84, 96.00) | 95.52 (94.14, 96.40) | 1.273 | 0.203 |
| FS | 86.97 (82.86, 88.83) | 88.59 (86.71, 90.69) | 2.637 | 0.008* |
| B | 14.98 (13.14, 16.90) | 14.19 (12.71, 15.35) | 3.169 | 0.002* |
| BN | 12.67 (11.04, 14.41) | 11.70 (10.28, 13.13) | 2.900 | 0.004* |
| BS | 17.80 (15.44, 21.40) | 15.34 (12.91, 18.10) | 4.010 | <0.001* |
| FG0 | 21.05 (19.49, 24.78) | 18.69 (15.02, 22.23) | 3.138 | 0.002* |
| FG1 | 18.09 (16.63, 20.83) | 19.48 (16.39, 22.40) | 1.980 | 0.048* |
| FG2 | 12.99 (9.76, 15.20) | 15.04 (12.58, 17.43) | 3.591 | <0.001* |
| aGal/Gal ratio | 38.97 (34.38, 50.31) | 32.82 (28.88, 43.19) | 3.482 | <0.001* |

*Statistically significant, *P* < 0.05. *P* values were calculated by the Mann‒Whitney U test. B, bisecting GlcNAc; F, core fucose; G, galactose; GP, glycan peak; LN, lupus nephritis; N, neutral glycans; S, sialic acid.

Table S5. Stepwise logistic regression-based screening of IgG N-initial glycan markers in LN

| Variables | *β* | *SE* | Wald | OR (95% CI) | *P* |
| --- | --- | --- | --- | --- | --- |
| **Model 1** |  |  |  |  |  |
| GP8 | -0.754 | 0.177 | 18.138 | 0.470 (0.333, 0.666) | <0.001** |
| GP10 | 0.436 | 0.157 | 7.777 | 1.547 (1.139, 2.103) | 0.005* |
| GP18 | -0.829 | 0.173 | 22.908 | 0.436 (0.311, 0.613) | <0.001** |
| Constant | 2.893 | 0.786 | 13.541 | - | <0.001** |
| **Model 2** |  |  |  |  |  |
| Anemia | 0.953 | 0.323 | 8.686 | 2.594 (1.376,4.890) | 0.003* |
| WBC | 0.12 | 0.054 | 4.934 | 1.128 (1.014,1.254) | 0.026* |
| Constant | -1.148 | 0.418 | 7.53 | - | 0.006* |
| **Model 3** |  |  |  |  |  |
| Anemia | 1.087 | 0.371 | 8.595 | 2.965 (1.434, 6.132) | 0.003* |
| GP8 | -0.805 | 0.184 | 19.039 | 0.447 (0.312, 0.642) | <0.001** |
| GP10 | 0.514 | 0.165 | 9.742 | 1.671 (1.211, 2.307) | 0.002* |
| GP18 | -0.822 | 0.179 | 21.136 | 0.440 (0.310, 0.624) | <0.001** |
| Constant | 2.442 | 0.819 | 8.896 | - | 0.003* |

*Statistically significant, *P < 0.05, **P < 0.01; *P* values were calculated by stepwise logistic regression. *β*, regression coefficient; CI, confidence interval; LCI, lower confidence interval; LN, lupus nephritis; OR, odds ratio; GP, glycan peak; *SE*, standard error; UCI, upper confidence interval.

Table S6. multivariate logistic regression-based identification model of LN

| Variables | *β* | *SE* | Wald | OR (95% CI) | *P* |
| --- | --- | --- | --- | --- | --- |
| **Model 4** |  |  |  |  |  |
| GP8 | 0.797 | 0.183 | 19.054 | 0.451 (0.315, 0.645) | <0.001** |
| GP10 | 0.423 | 0.157 | 7.230 | 1.526 (1.121, 2.077) | 0.007** |
| GP18 | 0.864 | 0.178 | 23.517 | 0.421 (0.297. 0.598) | <0.001** |
| uCRE | 0.010 | 0.022 | 0.230 | 1.010 (0.968, 1.054) | 0.632 |
| eGFR | 0.012 | 0.012 | 0.961 | 0.988 (0.965, 1.012) | 0.327 |
| Constant | 3.848 | 1.117 | 11.855 | - | 0.001** |
| **Model 5** |  |  |  |  |  |
| uCRE | 0.001 | 0.019 | 0.001 | 1.001 (0.964, 1.039) | 0.973 |
| eGFR | 0.003 | 0.010 | 0.103 | 0.997 (0.977, 1.017) | 0.748 |
| Constant | 0.332 | 0.500 | 0.440 | - | 0.507 |

*Statistically significant, **P* < 0.05, ***P* < 0.01; *P* values were calculated by multivariate logistic regression. *β*, regression coefficient; CI, confidence interval; LCI, lower confidence interval; LN, lupus nephritis; OR, odds ratio; GP, glycan peak; *SE*, standard error; UCI, upper confidence interval; uCRE, urine creatinine; eGFR, estimated glomerular filtration rate.

Table S7. Evaluation of the identification performance effect of a three-group model to distinguish between the LN and non-LN groups of SLE patients

| Models | Sensitivity | Specificity | Youden’s Index | AUC | 95% CI of AUC | |
| --- | --- | --- | --- | --- | --- | --- |
|  |  |  |  |  | LCI | UCI |
| Model 1 | 0.484 | 0.806 | 0.290 | 0.647 | 0.568 | 0.726 |
| Model 2 | 0.839 | 0.602 | 0.441 | 0.769 | 0.702 | 0.837 |
| Model 3 | 0.753 | 0.742 | 0.495 | 0.792 | 0.727 | 0.858 |
| Model 4 | 0.699 | 0.763 | 0.462 | 0.761 | 0.691 | 0.830 |
| Model 5 | 0.108 | 0.968 | 0.076 | 0.514 | 0.430 | 0.597 |

Model 1 consists of GP8, GP10, and GP18. Model 2 consists of anemia and WBC count. Model 3 consists of anemia, GP8, GP10, and GP18. Model 4 consists of Scr, eGFR, GP8, GP10, and GP18. Model 5 consists of Scr and eGFR. GP, glycan peak; AUC, area under the curve; CI, confidence interval; LCI, lower confidence interval; LN, lupus nephritis; ROC, receiver operating characteristic curve; UCI, upper confidence interval; SLE, systemic lupus erythematosus; eGFR, estimated glomerular filtration rate;.Scr, Serum creatinine.
